# Diagnostic Algorithm Using Multimodal Imaging for the Differential Diagnosis of Intra-Cardiac Masses

**DOI:** 10.3390/jcm14020508

**Published:** 2025-01-15

**Authors:** Fabiola B. Sozzi, Eleonora Gnan, Andrea Pandolfi, Laura Iacuzio, Jin Kyung Kim, Ciro Canetta, Alessandra S. Rizzuto, Massimiliano Ruscica, Stefano Carugo

**Affiliations:** 1 Department of Cardio-Thoracic-Vascular Diseases, Foundation IRCCS Ca’ Granda Ospedale Maggiore Policlinico, 20122 Milan, Italy; fabiola_sozzi@yahoo.it (F.B.S.); eleonora.gnan@gmail.com (E.G.); andreapandolfi96@gmail.com (A.P.); stefano.carugo@unimi.it (S.C.); 2 Cardiothoracic Centre CCM, 98000 Monaco, Monaco; l.iacuzio@ccm.mc; 3 Division of Cardiology, University of California, Irvine, CA 92697, USA; jkim13@hs.uci.edu; 4 High Care Internal Medicine Unit, Foundation IRCCS Ca’ Granda Ospedale Maggiore Policlinico, 20122 Milan, Italy; ciro.canetta@policlinico.mi.it; 5 Department of Clinical Sciences and Community Health, Università Degli Studi Di Milano, 20122 Milan, Italy; alessandra.rizzuto@unimi.it; 6 Department of Pharmacological and Biomolecular Sciences “Rodolfo Paoletti”, Università Degli Studi Di Milano, 20133 Milan, Italy

**Keywords:** cardiac masses, noninvasive imaging, transthoracic echocardiography, transesophageal echocardiography

## Abstract

Cardiac masses are complex clinical conditions that frequently pose diagnostic challenges in cardiology practice. These masses can form within heart chambers or near the pericardium and are generally categorized as either non-neoplastic or neoplastic. These latter are further classified into benign and malignant (primary and secondary or metastatic). Diagnosing these conditions often requires a multiparametric approach that includes both clinical features, such as the patient’s and associated clinical conditions, and multimodality imaging. However, histological examination of the resected specimen is often necessary to ascertain the nature of the mass. Given their heterogeneity and the rarity of many cardiac masses, there are no guidelines or consensus on the best diagnostic approach. Modern imaging protocols must be tailored to the specific clinical issues and patient characteristics, given the rapid advancements in technology. Thus, it is imperative to use a multimodality approach, combining different imaging techniques and multidisciplinary teamwork. Hereby, we propose a practical algorithm for evaluating cardiac lesions using a step-by-step implementation of multimodal imaging. Ultimately, the goal is to tailor the most suitable imaging technique to the patient’s needs.

## 1. Introduction

Cardiac masses are a broad set of space-occupying structures within the cardiac cavities or adjacent to the pericardium that remain a common diagnostic challenge in cardiology practice and can be broadly classified as neoplastic or non-neoplastic. The latter include frequently diagnosed clinical entities such as thrombi and vegetations, while neoplastic masses are comprised of metastases from extracardiac tumors and primary cardiac tumors (PCTs), both benign and malignant. Secondary metastases are by far the most frequent form of cardiac tumors and, by definition, malignant. PCTs, on the contrary, are exceedingly rare and mostly benign (90%). In adults, the most common histotypes of benign cardiac tumors include myxomas, lipomas, and papillary fibroelastomas (PFEs), while in the pediatric population rhabdomyomas and fibromas are by far more represented [1,2]. On the other hand, primary malignant cardiac tumors (such as sarcoma, angiosarcoma, rhabdomyoma, and lymphoma) are rare. The clinical presentation of cardiac tumors can be heterogeneous. Many masses are incidentally discovered during an echocardiographic examination or an imaging study performed for other reasons. Alternatively, patients may present with constitutional and paraneoplastic symptoms, such as fever, malaise, or weight loss. Metastases and malignant PCTs are often accompanied by pericardial effusion which, in severe cases, may result in cardiac tamponade. Intracardiac masses may interfere with valve function and heart filling or result in cardiac arrhythmias, possibly leading to dyspnea, chest pain, syncope, and hemodynamic impairment. Finally, many cardiac masses are prone to pulmonary or systemic embolization [3]. The differential diagnosis of cardiac masses requires careful consideration of factors such as prevalence, patient age, anatomical location, and associated clinical conditions.

## 2. Overview of Imaging Techniques

A multiparametric approach that considers both clinical features, such as age at presentations and associated clinical conditions, and multimodality imaging is often necessary to reach a diagnosis. Still, histological analysis of resected specimens is often required to ascertain the nature of the lesion ultimately. Transthoracic echocardiography (TTE) is the first-line imaging tool to evaluate cardiac masses, due to its wide availability, safety, and low cost. It is ideal for assessing small, mobile, or valve-related masses like vegetations and PFEs, providing insights into the size, morphology, mobility, and hemodynamic impact. Echocardiographic features suggesting possible malignancy include pericardial effusion, immobility, heterogeneous echogenicity, and myocardial invasion. Limitations of TTE include reduced accuracy in patients with poor acoustic windows (e.g., obesity) and difficulty in distinguishing mass composition. Transesophageal echocardiography (TEE) is frequently required to better define the size, shape, attachment, and relationship of the mass with adjacent structures. Moreover, contrast-enhanced echocardiography may be required to improve intracavitary definition or to exclude thrombus, especially in cases of poor acoustic windows. Real-time three-dimensional echocardiography (RT3DE), either transthoracic or transesophageal, represents a significant advancement in the evaluation of cardiac masses, offering unparalleled insights compared to traditional two-dimensional echocardiography. As a volumetric imaging technique, RT3DE captures the entire cardiac mass in three dimensions, allowing for accurate assessment of its size, volume, attachments, and spatial relationships with surrounding structures. RT3DE eliminates many limitations of two-dimensional echocardiography by providing direct volumetric measurements rather than extrapolations, improving diagnostic accuracy and interobserver consistency. Beyond diagnosis, RT3DE is also a valuable tool for intraprocedural guidance during surgical interventions, ensuring precise localization of attachment points and enhancing procedural outcomes [4,5].

Cardiac magnetic resonance (CMR) provides a comprehensive, multiplanar, and non-invasive morphological and histological evaluation of cardiac masses. It is regarded as the “gold standard” for non-invasive assessment of cardiac masses, owing to its unique ability to characterize tissues depending on their proton density and their T1 and T2 relaxation times. Perfusion characterization through the acquisition of T1-weighted (“black blood”), T2-weighted, fat-suppressed perfusion, early and late gadolinium enhancement (LGE) sequences can reveal adipose infiltration, increased vascularization, necrosis, fibrosis, increased water content or hemorrhage within the mass. Additionally, CMR allows detailed morphological definition of the mass, in terms of size, location, homogeneity, and its relationship with adjacent structures, and thus plays a pivotal role during pre-surgical planning [6]. Limitations of CMR include its limited availability, long acquisition times that make it problematic in the presence of hemodynamically unstable or claustrophobic patients, and lower spatial and temporal resolution compared to echocardiography, making it less optimal for assessing small lesions (<7 mm) [4].

Cardiac computed tomography (CCT) plays a supportive role in imaging diagnostics and serves as an alternative to CMR in cases where the latter is contraindicated or unavailable. Advantages of CCT include broad availability, fast acquisition times, and high spatial resolution, especially when evaluating thoracic structures. It is a valuable tool for analyzing mass characteristics and tissue composition and is fundamental during pre-operative planning. However, its resolution is less precise than echocardiography for very small formations, typically under 4 mm, and its temporal resolution is reduced as compared to CMR. Other limitations include suboptimal evaluation of valvular vegetation and the reliance on ionizing radiation and iodinated contrast agents. Nonetheless, it remains particularly adept at distinguishing intracavitary tumors from thrombi and excels in studying calcified masses, which CMR struggles to characterize accurately [4]. Finally, 18F-fluorodeoxyglucose positron emission tomography (18F-FDG-PET) provides an accurate assessment of tumor metabolic activity and is increasingly recognized as a third-line diagnostic tool for cardiac masses, particularly when combined with CT to improve spatial resolution. It is widely used in oncology for differentiating benign from malignant tumors, staging, guiding biopsies, planning radiotherapy, and monitoring treatment response. PET can also detect residual tumors post-surgery or incidental cardiac metastases in patients with extracardiac cancers [3,4]. Limitations of cardiac PET include exposure to radiation, preparation requirements, physiological myocardial uptake variability, limited availability, and difficulty distinguishing primary from metastatic cardiac masses. Inflammatory or infectious conditions and factors like glucose levels or insulin therapy may also affect diagnostic accuracy.

Indications, strengths, and limitations of each imaging technique are reported in Table 1.

## 3. Proposed Algorithm for the Diagnosis of Cardiac Masses

**Figure 1:**
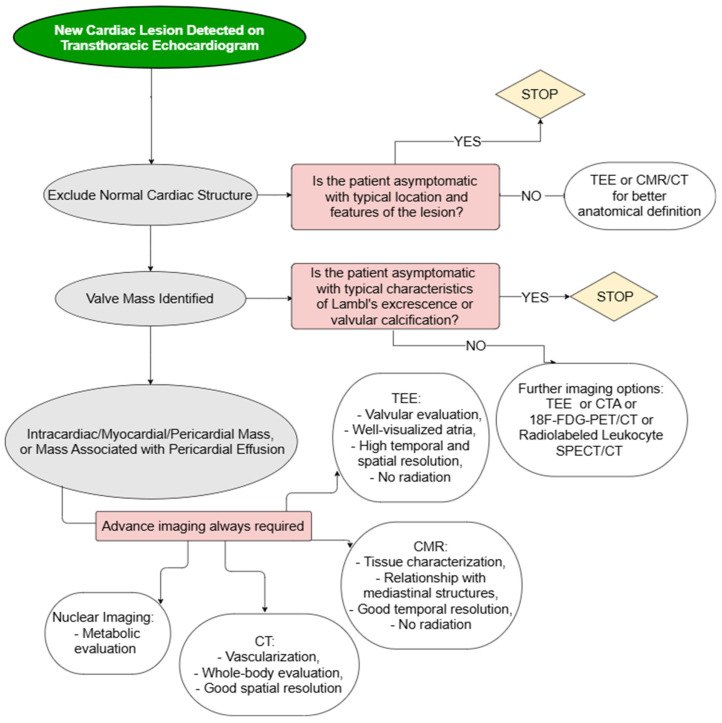
Proposed diagnostic algorithm. 18F-FDG-PET/CT:18-fluorodeoxyglucose positron emission tomography/computed tomography; CMR: cardiac magnetic resonance; CT: computed tomography; PFEs: papillary fibroelastomas; TEE: transesophageal echocardiography.

Given their heterogeneity and the rarity of many cardiac masses, there are no guidelines or consensus statements regarding the best diagnostic approach to undertake. Hereby, we propose a practical algorithm for diagnosing cardiac lesions with the stepwise implementation of multimodality imaging (Figure 1). In short, TTE may be sufficient to reasonably rule out suspicious lesions if the mass being evaluated displays typical features of a normal cardiac structure (e.g., crista terminalis, coumadin ridge) and the patient is asymptomatic in the absence of high risk associated findings on imaging. Similarly, a valvular mass demonstrating characteristics consistent with benign conditions such as Lambl’s excrescences or calcifications in an asymptomatic patient may not require further diagnostic tests. In all other scenarios, on the contrary, additional imaging is required to achieve an accurate diagnosis and provide appropriate management tailored to the lesion type and clinical context.

The application of the proposed algorithm for diagnosing cardiac masses may, however, present several challenges, including managing ambiguous findings and ensuring access to advanced imaging modalities. Normal anatomical variants may be misinterpreted as pathological, emphasizing the need for clinician training in echocardiographic anatomy, including its less common aspects. The frequent need to rely on advanced or multimodal imaging such as TEE, CMR, or PET/CT could pose a challenge due to the limited availability of these modalities, particularly in resource-constrained settings. Additionally, integrating clinical data, such as symptoms, embolic risk, and inflammatory markers with imaging findings is crucial for making an accurate diagnosis, but such data may not always be readily or easily available. These limitations may hinder adherence to the algorithm; nevertheless, its implementation would help reduce unnecessary testing, allowing for better allocation of resources where they are most needed, underscoring the importance of establishing clear referral pathways and prioritization frameworks. To ensure its reliability, the algorithm should be validated through registries with long-term follow-up of patients.

**Figure 2:**
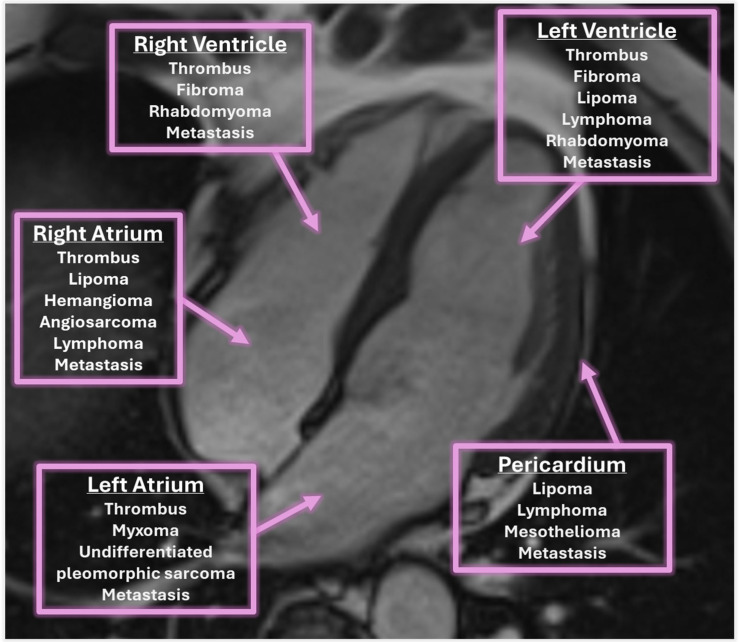
Localization of the most common cardiac masses.

A schematic representation of the most common masses and their localization is represented in Figure 2 and described throughout the text.

## 4. Pseudo-Masses: Anatomic Variants of Normal Intracardiac Structures

When evaluating an intracardiac mass, it is essential to determine if the mass is normal or a normal variant. Advanced imaging techniques, such as TEE, CCT, and CMR, would only be necessary for symptomatic patients, with atypical lesion locations or features.

### 4.1. Coumadin Ridge

The coumadin ridge, also known as warfarin or left lateral ridge, is an anatomical variant in the left atrium (LA), located between the left superior pulmonary vein and the left atrial appendage (LAA). It is a band-like fold that contains the ligament of Marshall, autonomic nerves, and small arteries. It often resembles a cotton-tip applicator (“Q-tip sign”) on a 2D imaging. While prominent ridges can mimic a thrombus or mass, the unique location of these structures is an important clue to proper identification. CMR can help visualize the ridge, with contrast echocardiography sometimes used to rule out thrombi or tumors [7].

### 4.2. Crista Terminalis

The crista terminalis is a smooth muscular ridge located in the upper right atrium (RA), marking the boundary between the smooth-walled sinus venarum (derived from the embryologic sinus venosus) and the more trabeculated right atrial appendage. It runs between the openings of the superior and inferior vena cava and is localized along the posterolateral wall of the RA. On TTE, it can sometimes appear as a mass but features like its rounded margins, similarity in echogenicity to the myocardium, and size changes during atrial contraction help differentiate it from other causes [8]. It is best seen in the apical four-chamber view, while during TEE the bicaval view offers a detailed look at its relationship to adjacent structures. Furthermore, it is easily recognized by CMR.

### 4.3. Chiari Network

The Chiari network is a web-like structure in the RA, resulting from incomplete resorption of the embryonic sinus venosus, which also forms the Eustachian valve. On echocardiography, it appears as a reticulated, hypermobile membranous structure. It typically extends from the posterior RA, extending medially and inferiorly, to the interatrial septum. It moves freely during systole and diastole, does not migrate into the right ventricle (RV), and is often tethered near the inferior vena cava, where it may seem to rotate [9].

### 4.4. Moderator Band

The moderator band, also known as the septomarginal trabecula, is a consistent structure in the RV and serves as a useful landmark when distinguishing between the ventricles, especially in cases of congenital heart disease. The name “septomarginal” describes its course, as it extends from the interventricular septum to the margin of the RV, where it connects to the base of the anterior papillary muscle. The moderator band is not involved in valve function but plays a role in the heart’s electrical conduction system as part of the right bundle branch [10].

### 4.5. Lipomatous Hypertrophy of the Interatrial Septum

Lipomatous hypertrophy of the interatrial septum is a benign condition characterized by fatty infiltration of the interatrial septum, typically found in elderly or obese patients as an incidental, asymptomatic finding. On echocardiography, it appears as a well-defined thickening of the interatrial septum, usually exceeding 2 cm, with a distinctive dumbbell shape due to the sparing of the fossa ovalis [11].

CMR can confirm the diagnosis by showing bilobar septal thickening with signal intensity similar to subcutaneous fat (T1 and T2: high signal, T1-fat suppressed with low signal and no LGE). PET-CT may show moderate FDG uptake, initially thought to be related to brown adipose tissue, though this is debated, with inflammation or other mechanisms also considered [12].

### 4.6. Cardiac Blood Cyst

Cardiac blood cysts (BCs) are rare, benign cardiac masses that may arise congenitally or be acquired post-surgery or due to other cardiac conditions. They commonly attach to valvular structures, particularly the mitral and tricuspid valves, and less frequently involve other cardiac regions. BCs vary in size (typically 4–30 mm) and shape (round, oval, or multilobular) and exhibit an anechoic, thin-walled, and mobile appearance on echocardiography [13]. Although BCs are often asymptomatic, they can cause symptoms such as dyspnea, palpitations, and cyanosis. Complications include valve prolapse, obstruction of cardiac orifices, and systemic embolism. Surgical resection is recommended for symptomatic cases or those at risk of complications like embolism, while asymptomatic BCs may be monitored with serial imaging. Advanced imaging modalities play a critical role in diagnosis and management. Contrast-enhanced echocardiography can confirm the absence of vascular perfusion and identify fine anatomical details such as stalks or attachments. CMR imaging provides additional insights, characterizing the cyst’s composition and ruling out malignant or thrombotic masses. Similarly, cardiac CT is effective in detecting calcifications, cystic walls, and anatomical relationships, particularly in patients with contraindications to CMR. TEE enhances visualization of smaller or posteriorly located cysts, improving diagnostic accuracy for subtle lesions.

## 5. Intracardiac and Myocardial Masses

### 5.1. Non-Tumoral Masses

#### 5.1.1. Intracardiac Thrombi

Thrombus is the most common type of cardiac mass and distinguishing it from a cardiac tumor can be complex, as they may present similar imaging features. Detecting thrombi is crucial to prevent embolic events and to guide anticoagulation therapy. They are typically found in the LAA in patients with atrial fibrillation, atrial enlargement, mitral stenosis, or a history of mitral valve surgery. After a myocardial infarction (MI), a thrombus might form next to a hypokinetic or akinetic region of the left ventricle (LV), commonly at the apex in cases of anterior MI, with or without associated wall aneurysm. Less frequently, thrombi can occur in the RA or in the inferior vena cava, usually in individuals with right-sided chamber enlargement or central venous lines. Echocardiography is the preferred initial diagnostic tool for detecting thrombi, which usually have a broad base and are immobile [14]. Acute thrombi tend to be hypoechoic, while more chronic masses exhibit higher echo density or more complex echotexture. Thrombi in the LAA require a transesophageal approach for accurate detection. LV apical thrombi can be difficult to visualize on a TTE, and when needed, contrast echocardiography should be utilized to confirm the diagnosis, showing a dark intracavitary mass without contrast uptake.

On CCT, they appear as low-attenuation areas on delayed phase images. CMR is the most effective technique to differentiate between thrombi and cardiac tumors, with T1- and T2-weighted signal intensities varying by thrombus age. In the acute phase, they appear iso- to hyperintense on T1- and T2-weighted images, while chronic thrombi are hypointense on both. Most notably, thrombi lack contrast enhancement during first-pass perfusion, early imaging phases, and LGE imaging [15]. However, large, chronic thrombi may occasionally exhibit peripheral enhancement due to endothelization of the thrombus surface, making differentiation from tumors more challenging.

#### 5.1.2. Myocardial Calcifications

**Figure 3:**
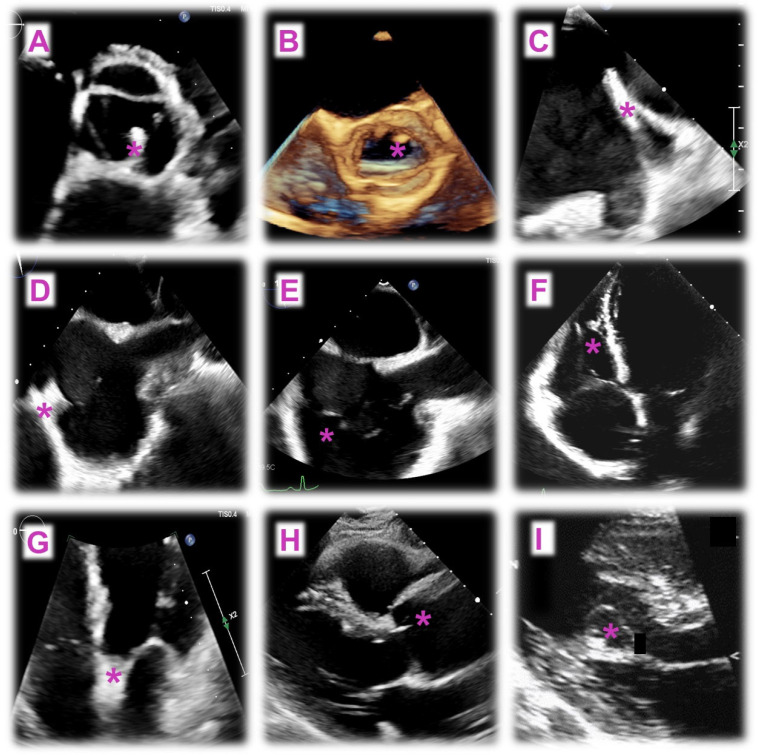
Echocardiographic images of masses and pseudomasses (*). Panel (**A**,**B**): Papillary fibroelastoma on TEE (**A**) and RT3DE (**B**); Panel (**C**): Coumadin ridge on TEE; Panel (**D**): Crista terminalis on TEE; Panel (**E**): Chiari network on TEE; Panel (**F**): Moderator band on TTE; Panel (**G**): Lipomatous hypertrophy of the interatrial septum on TEE; Panels (**H**): Lambl’s excrescences on a long-axis parasternal view, TTE; Panels (**I**): Cardiac blood cyst on TTE.

**Figure 4:**
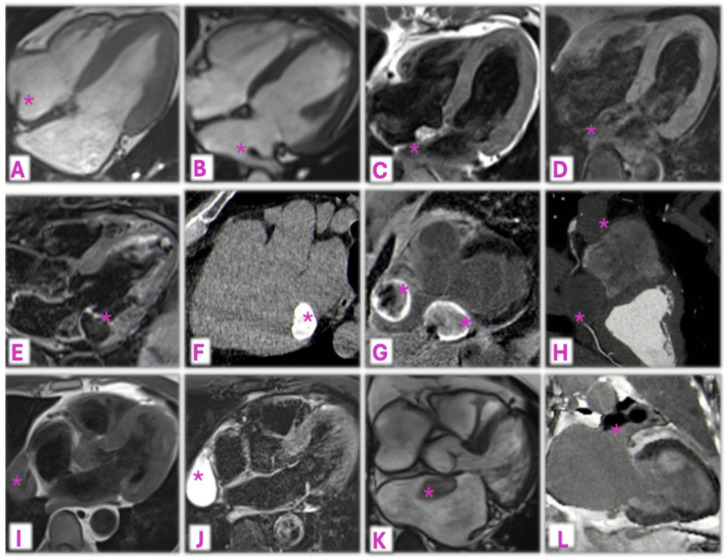
Examples of normal anatomical structures and non-tumoral masses at CMR/CCT (*). Panel (**A**): Crista terminalis; Panel (**B**): Coumadin ridge; Panels (**C**,**D**): Lipomatous hypertrophy of the interatrial septum showing hyperintense signal in turbo-spin-echo T1-weighted images (**C**) which notably decreases with fat saturation (**D**); Panels (**E**,**F**): Caseous mitral annular calcification has low signal on T2-weighted CMR images (**E**) and increased density on CCT (**F**); Panels (**G**,**H**): Two thrombosed aneurysms of the right coronary artery showing no-contrast Gadolinium uptake on CMR (**G**) and no iodine uptake on CCT (**H**); Panels (**I**,**J**): pleuro-pericardial cyst appearing homogeneously isointense in turbo-spin-echo T1 (**I**) and hyperintense in T2-weighted CMR images (**J**); Panels (**K**,**L**): examples of intracardiac thrombi, in the LA on cine-SSFP imaging (**K**) and in the LAA showing no-contrast uptake in LGE imaging (**L**).

Myocardial calcifications are a relatively uncommon pathological phenomenon that can be categorized as metastatic or dystrophic based on the underlying cause. Dystrophic calcifications, the most frequent type (70%), are a consequence of local cardiac cell injury and necrosis and result from conditions like endomyocardial fibrosis, MI, or sepsis. On the contrary, metastatic calcifications are linked to systemic disorders and disturbances in calcium metabolism, most commonly due to chronic kidney disease [16]. On echocardiography, myocardial calcifications typically appear as an area of increased echogenicity with posterior acoustic shadowing. CCT is considered the gold standard to accurately identify, localize, and assess the extent of calcifications, which appear as masses of increased density. Finally, on CMR, calcifications show low signal intensity on both T1- and T2-weighted images, with no LGE in the calcified areas but potential LGE in surrounding necrotic tissue. Examples of cardiac masses and pseudomasses as evaluated by echocardiography and CMR/CCT are represented in Figure 3 and Figure 4, respectively.

### 5.2. Cardiac Tumors: Benign

#### 5.2.1. Myxoma

Cardiac myxoma are the most common type of benign primary heart tumors, often occurring between the fourth and sixth decades of life, with a slight predominance in females. They are mostly located in the LA (over 80% of cases) though, especially in children, it may also arise in the other chambers. They typically appear as intracavitary masses attached by a stalk to the fossa ovalis but can also be found in other regions such as the atrial free wall or mitral valve leaflets. Most occur sporadically, but in 3–10% of cases, they are associated with familial syndromes, particularly Carney complex, an autosomal dominant genetic disorder characterized by endocrinopathies and spotty skin pigmentation [17,18]. Polypoid myxomas, when large, can cause obstruction and may produce a distinctive “tumor plop” sound during auscultation, while papillary myxomas are more likely to cause embolic events. Additionally, constitutional symptoms like fatigue, fever, and weight loss have been reported. Complete surgical resection is the preferred treatment for cardiac myxomas, providing both a definitive diagnosis and the best chance of preventing major complications. However, recurrence occurs in 10–15% of cases, most commonly at the site of the original tumor. Myxomas typically present as mobile polypoid or papillary masses attached to the atrial septum by a stalk, with their movement influenced by the stalk’s length and the tumor’s shape. These masses are often globular or spherical, with a fragile lobulated surface and heterogeneous echogenicity. Contrast-enhanced echocardiography aids in providing clearer visualization [19]. TEE can assist in visualizing the tumor’s attachment site and identifying any potential extension into the pulmonary or caval veins. On CCT, myxomas typically appear as well-defined, low-attenuation intracavitary masses with lobulated or slightly villous surfaces, sometimes showing heterogeneous enhancement during delayed phase imaging. Calcification is seen in about 14% of cases, more commonly in right-sided lesions. CMR usually shows the tumors as isointense on T1-weighted images and hyperintense on T2-weighted images, reflecting their high water content. LGE imaging shows heterogeneous enhancement due to cystic degeneration, necrosis, hemorrhage, calcification, and associated thrombus, and steady-state free precession imaging can reveal tumor prolapse through the mitral or tricuspid valves, helping identify the stalk’s attachment. Additionally, native T1 and T2 relaxation times, along with extracellular volume values, are elevated compared to normal myocardium, reflecting increased fluid content and interstitial space [20,21].

#### 5.2.2. Lipoma

Cardiac lipomas are rare benign tumors, comprising 3–12% of primary benign cardiac tumors, most often found in middle-aged and older adults. Composed of mature adipose tissue, they can arise from subendocardial (often in the RA), subepicardial, or myocardial layers, and are typically asymptomatic, though they may occasionally cause arrhythmias, valvular dysfunction, or coronary artery compression. Surgical removal is reserved for symptomatic cases (3). On TTE, lipomas appear as well-defined, broad-based, homogeneous immobile masses without a stalk. They appear hyperechoic in the heart chambers, and hypoechoic in the pericardium, and typically show no calcification on CMR. They appear uniformly hyperintense in both T1- and T2-weighted images, with low signal intensity in fat-saturated sequences. On cine-MRI, they may demonstrate the so-called “india ink” artifact, also known as black boundary or type 2 chemical shift artifact. Their benign characteristics and lack of blood supply are further supported by the absence of LGE. CCT imaging further identifies them as homogeneous, low-attenuation masses resembling fat [21,22].

#### 5.2.3. Other Rare Benign Tumors: Rhabdomyomas, Fibromas, Hemangiomas

Rhabdomyomas are the most common PCTs in children, typically detected in utero or within the first year of life. Around 70% are linked to tuberous sclerosis, which often presents as multiple masses and is characterized by the triad of seizures, intellectual disability, and facial angiofibromas. They are primarily located in the ventricles and can be either intramyocardial or intracavitary. Most spontaneously regress during early childhood and surgical intervention is only necessary for significant obstruction or persistent arrhythmias. On TTE or fetal echocardiography, they appear as small, well-circumscribed, and bright multiple nodules or pedunculated masses, protruding into the ventricles or embedded in the myocardium. On CMR, they are isointense on T1 but hyperintense on T2-weighted images, with minimal LGE. CCT typically shows these lesions as homogeneous, low-attenuation intramural masses with potential intracavitary extension [23,24].

Cardiac fibromas are the second most common pediatric tumors and are often diagnosed within the first year of life. They are typically solitary masses located in the ventricular free wall or interventricular septum, and a small percentage is linked to Gorlin syndrome. While benign, they may interfere with the conduction pathway or lead to ventricular arrhythmias and sudden death. Due to the associated arrhythmic risk and the lack of spontaneous regression, surgical resection is recommended regardless of symptomatology. On TTE, fibromas typically present as well-defined, solid, highly echogenic masses within the myocardium, and may display areas of central calcification. At CMR, they exhibit iso-hypointensity on T1 and hypointensity with a possible hyperintense peripheral rim on T2-weighted images. They appear hypointense on first-pass perfusion but display high LGE, probably related to their fibrotic nature. CCT usually reveals an intramyocardial, homogeneous, low-attenuation mass with central calcifications, variable margins, and minimal to no contrast enhancement [25].

Cardiac hemangiomas are rare primary tumors mostly seen in the fifth decade. They are characterized by benign proliferative endothelial cells lining vascular channels. They are typically solitary and can arise anywhere in the heart, even at the level of the valves or pericardium, with the RA being the most common site. Patients are often asymptomatic, but life-threatening obstruction, embolism, or arrhythmias may also occur, and, for this reason, surgical excision is often recommended despite their benign nature, especially for symptomatic patients. On echocardiography, hemangiomas typically appear as echogenic vascular masses of 2 to 5 cm. CMR and CCT imaging are particularly useful to show their high vascularity, while coronary angiography can also be used to identify feeding vessels associated with the tumor. Finally, PET has been used in a limited number of cases, primarily to differentiate cardiac hemangiomas from metastatic tumors [26].

### 5.3. Cardiac Tumors: Malignant

#### 5.3.1. Primary Cardiac Sarcoma

Cardiac sarcomas represent more than two-thirds of all malignant PCTs and can be associated with Li–Fraumeni syndrome, a rare autosomal dominant disorder characterized by early onset of cancer. Compared with extracardiac soft tissue sarcomas, cardiac sarcomas are seen in younger patients in their 40s, except for rhabdomyosarcomas which represent the most common pediatric cardiac malignancy. Surgery and palliative chemotherapy are the mainstay of treatment, even if most tumors have already metastasized at the time of diagnosis and carry a dismal prognosis, with a 5-year survival rate of 14% [27]. The most common histopathological subtypes include angiosarcomas, undifferentiated pleomorphic sarcomas, leiomyosarcomas, and rhabdomyosarcomas [28]. Angiosarcomas mainly originate in the RA, and rhabdomyosarcomas in the right chambers, while undifferentiated pleomorphic sarcomas and leiomyosarcomas have a predilection for the LA. On echocardiography, sarcomas appear as bulky, echogenic, broad-based irregular masses, which tend to invade and engulf the adjacent myocardial wall and surrounding structures and are often accompanied by pericardial effusion. A CT scan shows irregular intracavitary lesions with necrotic areas and low attenuation, while on CMR they typically appear heterogeneously isointense on T1 and hyperintense on T2-weighted images, with heterogeneous LGE [1,2,3]. 

#### 5.3.2. Primary Cardiac Lymphoma

Primary cardiac lymphomas (PCLs) are rare, aggressive extranodal lymphomas, mostly diffuse large B-cell types, often arising in the epicardial lymphatic network. PCLs commonly affect older, immunocompromised individuals, whereas secondary cardiac involvement from extracardiac lymphomas is more common, affecting roughly 25% of lymphoma patients. Clinical presentation is nonspecific and includes constitutional symptoms, chest pain, heart failure, and arrhythmias. Diagnosis relies on cytology of pericardial effusions or histology, and treatment involves anthracycline-based chemotherapy with rituximab. Despite good initial responses, recurrence is frequent, often in extranodal sites, and prognosis remains poor [29]. TTE typically demonstrates homogeneous, infiltrative masses that cause wall thickening and restrictive physiology, or as nodular masses protruding into the right heart chambers, especially the RA. The atrioventricular groove may also be involved, often encasing the right coronary artery, and the pericardium may show effusion and/or thickening. CMR imaging findings are consistent between PCLs and secondary lymphomas, which appear homogeneous, iso- to hypointense on T1, and mildly hyperintense on T2-weighted images due to diffuse edema and showing minimal or no contrast enhancement. Dense cellularity in lymphomas generally results in marked diffusion restriction. On CCT, they appear as large focal or infiltrative masses, or as multiple nodules with heterogeneous enhancement, often accompanied by mediastinal lymphadenopathy [30]. Finally, PCLs exhibit high metabolic activity on PET imaging.

#### 5.3.3. Secondary Cardiac Tumors

Cardiac metastases are the most prevalent type of malignant tumor involving the heart, with post mortem findings revealing heart or pericardial metastasis in up to 12% of cancer patients, though these are often asymptomatic. They generally occur in the pericardium (about 60%), while less frequently involve the epicardium and myocardium (each around 20%). Cancers with the highest propensity for cardiac involvement include lung mesothelioma, melanoma, lung and breast carcinomas. Ovarian, gastric, and lymphoproliferative neoplasms have also shown high rates of cardiac extension. Malignant cells can spread to the heart via four main pathways: through the bloodstream, lymphatic system, venous extension, or by direct invasion from adjacent structures. Malignancies originating from thoracic structures tend to spread through direct extension or retrograde lymphatic diffusion, leading to pericardial lesions and malignant effusion. Melanoma often affects the myocardium via hematogenous spread, while transvenous extension with inferior vena cava thrombosis and growth in the RA can be seen in hepatocellular and renal cell carcinomas [31]. Cardiac metastases can manifest as pericardial effusion, tamponade, conduction disturbances, and heart failure due to myocardial replacement or valvular dysfunction. Although cardiac metastasis usually indicates advanced disease and poor prognosis, surgical intervention may be considered when symptoms of obstruction or specific hemodynamic issues cannot be managed medically. On echocardiography, cardiac metastases display a wide range of structural patterns that vary according to tumor type, location, and mode of invasion. Pericardial metastases may be accompanied by fibrin blood effusion and often present as multiple nodules or masses, but diffuse infiltration may also occur. Myocardial metastases may affect any chamber and, when large, result in adjacent cardiac compression and valvular obstruction with reduced flow. Pericardial effusion, possibly hemodynamically significant, is also frequently present [31]. CMR displays lesions with low signal intensity on T1 and high signal intensity on T2-weighted images. However, metastatic melanoma and hemorrhagic lesions stand out, appearing hyperintense on T1-weighted imaging due to the T1-shortening effects of melanin and blood breakdown products. Finally, 18F-FDG-PET will show high tracer uptake [32,33].

**Figure 5:**
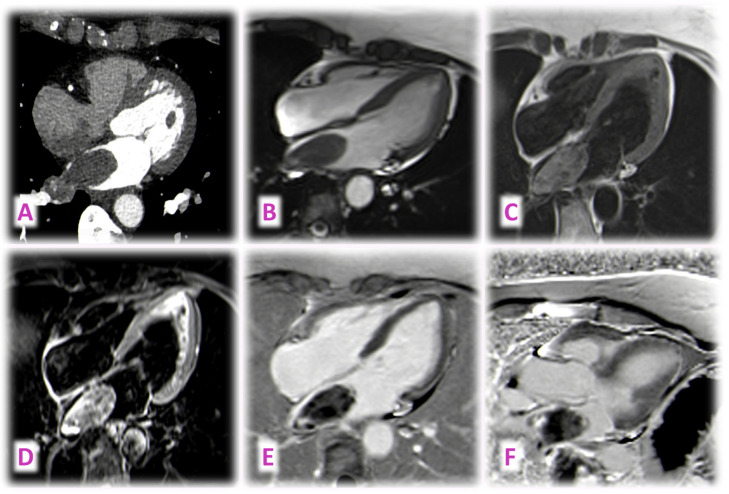
Cardiac CMR and CCT describe a mass at the level of the posterior LA/right pulmonary veins. Further histology demonstrated that it was a cardiac metastasis of a renal carcinoma.

We describe the case of a 68 y/o woman who presented for dyspnea. The TTE showed a mass in the LA originating from the right pulmonary vein. The cardiac CMR and CCT described a cardiac metastasis of renal carcinoma, at the level of the posterior LA/right pulmonary veins (Figure 5). The mass appears inhomogeneous on CCT (panel A) and CMR SSFP imaging (panel B), isointense in T1 (panel C), and hyperintense, in T2-weighted imaging (panel D), with heterogenous early (panel E) and LGE (panel F).

## 6. Differential Diagnosis of Intracardiac Masses

The differential diagnosis of cardiac masses requires careful consideration of factors such as prevalence, patient age, anatomical location, and associated clinical conditions. Thrombi are the most common cardiac masses overall, while metastases represent the most frequent cardiac neoplasms. Certain masses are more prevalent in specific age groups; for example, rhabdomyomas, fibromas, and rhabdomyosarcomas are more common in infants. Clinical context also provides crucial clues: thrombi are often linked to atrial fibrillation or recent MI, while immunodeficiency increases the likelihood of lymphoma. Additionally, some masses occur in inherited syndromes, such as myxomas in the Carney complex or rhabdomyomas in tuberous sclerosis. Indeed, anatomical location often hints at diagnosis. Myxomas typically arise from the LA with a stalk attached to the fossa ovalis and have a heterogeneous appearance. Lipomas, conversely, are more common in the RA, are homogeneous, and lack a stalk. Multiple masses in children suggest rhabdomyomas rather than fibromas. Intracardiac thrombus, which represents the major differential diagnosis for myxomas and other neoplasms, can be easily differentiated based on a complete absence of both early and late enhancement on CMR. However, it is not uncommon for a myxoma to present a layer of surface thrombus that typically shows a low signal on LGE images [21]. Features suggestive of malignancy include size >5 cm, broad-based implantation, irregular margins, right-heart involvement, invasion of adjacent structures, hemorrhagic pericardial effusion, heterogeneous MRI signals (indicative of necrosis or calcification), presence of first-pass perfusion and high LGE [20]. Several multiparametric scores have been developed to assess the probability of malignancy of a mass on non-invasive imaging. Among them, the CMR mass score [34] including sessile appearance, polylobate shape, infiltration, pericardial effusion, first-pass perfusion, and heterogeneous enhancement, showed excellent accuracy in predicting malignancy [areas under the curve, 0.976 (95% CI, 0.96–0.99)]. Moreover, a score of ≥5 predicted a higher risk of all-cause death at follow-up.

A study by Pazos-López et al. [35] on 116 patients highlights the utility of CMR in differentiating cardiac thrombi from tumors and benign from malignant neoplasms. Thrombi appeared smaller, homogeneous, and immobile, while tumors were larger, more heterogeneous, and motile, though this did not reliably distinguish benign from malignant. Signal intensity on T1 did not differ significantly, but hyperintensity on T2-weighted images was more common in tumors. Thrombi typically lacked first-pass perfusion or LGE, with LGE being more frequent in malignant than benign neoplasms. A post-contrast inversion time (TI) scout pattern showing hyperintensity/isointensity with short TI and hypointensity with long TI had 95% accuracy for identifying thrombi, which also displayed a distinct “edged” appearance with a dark rim and central bright zone at intermediate TI.

Finally, cardiac conditions like hypertrophic cardiomyopathy can mimic tumors such as fibromas but are distinguishable through T2-weighted imaging (in which fibromas appear hypointense) and CMR tagging, which highlights the contractile activity of the hypertrophied region [28].

The main CMR features of intracardiac masses are summarized in Table 2.

## 7. Valvular Masses

### 7.1. Papillary Fibroelastoma

PFEs are rare, benign cardiac tumors, with an incidence of less than 0.1%. They are mostly found in elderly patients and are slightly more common in males. PFEs typically arise from the valvular endocardium of the aortic valve, though other valves can also be involved. While often asymptomatic and incidentally discovered, they can lead to serious complications like stroke, MI, and peripheral embolization. Treatment involves surgical excision, especially for symptomatic patients or those with large (>9 mm) or highly mobile masses. After careful multidisciplinary discussion and exclusion of subclinical embolism with cerebral magnetic resonance, asymptomatic patients may be monitored, and anticoagulation can be considered for poor surgical candidates. TTE is the first-line imaging technique for diagnosing PFEs, with sensitivity and specificity above 85% for tumors larger than 2 mm. PFEs typically appear as mobile, pedunculated masses with a speckled edge pattern, ranging in size from 2 to 40 mm. They may arise from either the aortic or ventricular surfaces and exhibit independent movement. TEE is usually required when transthoracic results are inconclusive or for preoperative planning. Finally, advanced imaging may be needed to differentiate PFEs from other masses [36].

### 7.2. Lambl’s Excrescences

Lambl’s excrescences are thin, hypermobile strands that develop at the coaptation sites of cardiac valves. Their prevalence increases with age, peaking between ages 61 and 70 and declining thereafter due to valve calcification, which may hide them on imaging. They are more common in men and are found more frequently on left-sided valves, especially on the aortic valve. These growths are generally asymptomatic but can occasionally lead to serious complications, including embolic stroke, acute coronary syndromes, and pulmonary embolism. Treatment strategies include observation for asymptomatic cases and the use of antiplatelet agents or anticoagulation in patients with cryptogenic stroke. Surgical excision may be considered for recurrent embolic events, though there are no evidence-based guidelines. On TTE, Lambl’s excrescences appear as thin, filamentous, hypermobile strands of 1–10 mm in size, located on the endocardial surface where valvular cusps contact one another. Occasionally, multiple neighboring excrescences may cluster together, resulting in “giant Lambl’s excrescences” that can reach lengths of up to 2 cm. In a short-axis view, they appear as projections from the ventricular aspect of the aortic valve cusp tips, while in a long-axis view, they can be seen on all three aortic valve leaflets. When TTE results are inconclusive or inadequate, TEE serves as the gold standard for detecting and confirming the diagnosis. High-resolution CCT can also be used when TEE is not feasible [37].

### 7.3. Infective Endocarditis

Infective endocarditis (IE) is an inflammation of the heart’s inner lining and valves, primarily caused by bacteria, with an incidence of 3 to 10 cases per 100,000 people annually. It predominantly affects males, and the average age is over 65 years. Risk factors include the presence of prosthetic devices, immunosuppression, intravenous drug use, poor dental health, and degenerative valve disease. Clinically, IE may present with constitutional symptoms or symptoms related to valve dysfunction or distal embolization. Diagnosis relies on microbiological and imaging evidence, with treatment focused on prolonged antibiotic regimens and surgical intervention for severe cases or complications [38]. TTE is the first-line imaging method and shows vacillating intracardiac masses attached to the atrial side of atrioventricular valves, to the ventricular side of semilunar valves, or devices, often associated with new-onset valvular dysfunction. Implementation of TEE is recommended in almost all cases to confirm the diagnosis, evaluate perivalvular complications, and for surgical planning [39]. CCT excels in identifying paravalvular and periprosthetic complications, such as abscesses, pseudoaneurysms, and extracardiac embolic events. The use of CMR remains uncertain, while cerebral MRI is recommended for patients with neurological symptoms or those scheduled for valve surgery [40]. Nuclear imaging, particularly 18-fluorodeoxyglucose positron emission tomography/computed tomography (18F-FDG-PET/CT) and radiolabeled leucocyte SPECT/CT scintigraphy, plays a crucial role in diagnosing IE. The former allows early detection of infection before structural damage occurs, while the latter is useful when echocardiography is inconclusive, especially in the early postoperative phase. Both methods excel in detecting extracardiac infection foci [39,41].

### 7.4. Nonbacterial Thrombotic Endocarditis

Nonbacterial thrombotic endocarditis (NBTE), also known as Libman–Sacks or marantic endocarditis, is a rare condition associated with hypercoagulable states such as malignancy and autoimmune disorders. Patients are often asymptomatic, and NBTE is commonly discovered incidentally or postmortem. The vegetations are sterile masses made of fibrin and platelets which may undergo peripheral embolization. Management typically focuses on treating the underlying condition and may involve anticoagulation for thromboembolic prevention. Surgery may be warranted in cases of significant valvular dysfunction, with a focus on preventing recurrent embolization [42]. On TTE vegetations are usually small (<10 mm), sessile masses with irregular borders and heterogeneous echogenicity, primarily located on the mitral and aortic valves. TEE is recommended when TTE results are inconclusive. Echocardiography, however, cannot differentiate vegetation caused by NBTE from those caused by IE. As such, laboratory tests, blood cultures, and nuclear imaging are necessary to exclude IE [43].

### 7.5. Caseous Mitral Annular Calcification and Calcified Amorphous Tumor

Caseous mitral annular calcification (CMAC) is a rare form of degenerative mitral annular calcification (MAC), affecting about 0.6% of MAC cases, typically in older patients. It features a “toothpaste-like” liquefied core of calcium, cholesterol, and fatty acids, surrounded by a calcified rim, and is associated with hypertension, chronic kidney disease, or disorders of calcium–phosphate metabolism. While generally benign, large CMACs can infiltrate the myocardium, and cause valve dysfunction, obstruction, embolization, or heart block. Differentiating CMAC from other masses near the mitral annulus can be challenging and often requires multimodality imaging. On TTE and TEE, CMAC appears as a well-defined, echo-dense mass with central echolucency and a calcified rim. CCT confirms its calcified nature, showing a hyperdense mass with hypodense central content and no contrast enhancement. CMR identifies a hypointense mass on T1- and T2-weighted sequences, with no first-pass perfusion enhancement but potential peripheral LGE [44,45].

Calcified amorphous tumors (CAT) are a rare non-neoplastic cardiac mass, typically arising from the mitral valve and annulus in the context of MAC, although they may arise from any ventricular chamber. Histologically, the mass consists of nodules of calcium in the background of degenerating amorphous fibrinous material. While most cases of CAT are benign and asymptomatic, they may pose a risk for embolic stroke. Surgical resection is therefore often considered, especially if the lesion is large or symptomatic [46]. On TTE, CAT typically appears as a large, round, mobile, and calcified mass attached to the ventricular side of the mitral annulus in the context of a MAC. Further diagnostic imaging, such as CCT and CMR, are often necessary to better define the tissue composition of the mass.

### 7.6. Differential Diagnosis of Valvular Masses

Differentiating PFEs, Lambl’s excrescences, IE, and NBTE require careful consideration of their unique characteristics. PFEs are typically larger than Lambl’s excrescences, often located away from the valvular closure line, and more prone to embolization. In contrast, Lambl’s excrescences are small, filiform, and usually located along the valvular closure line, presenting often as benign incidental findings. NBTE is mainly associated with systemic lupus erythematosus and antiphospholipid syndrome and features rounded, non-pedunculated thrombotic vegetations that can appear anywhere on the valve surface, typically without infection. Infective endocarditis, instead, presents with irregular vegetations that have a distinct echogenicity and are often accompanied by clinical signs of infection, predisposing conditions, and potential valvular dysfunction or dehiscence. Peripheral calcifications and avascularity are key features in CMACs, which can be used to differentiate CMACs from other masses.

## 8. Pericardial Masses and Masses Associated with Pericardial Effusion

### 8.1. Malignant Pericardial Effusion and Pericardial Metastasis

Malignant pericardial effusion is a common complication in advanced cancers, significantly impacting patient survival. While pericardial effusion typically arises in patients with an established cancer diagnosis, in some instances, it can be the initial sign of malignancy. Symptoms of malignant pericardial effusion vary, ranging from mild discomfort and dyspnea to severe hemodynamic compromise. Rapid diagnosis and timely treatment are essential due to the condition’s potential to escalate quickly into critical situations like cardiac tamponade. Pericardial effusion in cancer patients can result from several malignancies, including lung, breast, and esophageal cancers, as well as hematological cancers. Less common causes include PCTs like mesotheliomas, hemangiomas, and neurofibromas. The effusion develops via direct local extension of the tumor, metastatic spread through lymphatic or hematogenous routes or lymphatic obstruction. Primary pericardial tumors, though rare, also contribute to the development of pericardial effusion. The underlying mechanisms and origins of the malignant cells are crucial to understanding the disease’s progression and ensuring appropriate management. Imaging plays a crucial role in diagnosing and managing malignant pericardial effusion. Echocardiography is the primary imaging modality, essential for determining the size of the effusion, tracking its progression, and detecting cardiac tamponade. Serial echocardiographic evaluations are recommended in oncological patients, especially during and after treatments such as chemotherapy and radiation. CCT and CMR further aid in evaluating pericardial fluid and identifying any associated thoracic tumors. In cases of loculated or hemorrhagic effusions, or when pericardial thickening is suspected, both CCT and CMR offer superior diagnostic capabilities. A definitive diagnosis often requires histopathological evaluation of pericardial fluid or of the lesion itself [47].

### 8.2. Pericardial Cyst

Pericardial cysts, rare congenital anomalies affecting 1 in 100,000 people, arise from incomplete embryonic development of the pericardium or, less commonly, are acquired after trauma or surgery. They generally contain clear fluid and have an average size of a few centimeters. Typically, asymptomatic and found incidentally, they may occasionally cause chest pain or dyspnea due to compression, while life-threatening complications such as tamponade rarely occur. Management is usually conservative with regular echocardiographic monitoring. Symptomatic or enlarging cysts may require interventions such as percutaneous aspiration, ethanol sclerosis, or surgical resection. On TTE they appear as anechoic, thin-walled structures adjacent to the heart, often at the right cardiophrenic angle. CCT without contrast is the preferred modality for precise localization, showing a single, non-enhancing, thin-walled, ovoid homogenous mass without solid components. CMR offers superior tissue characterization and helps evaluate the compressive effects of the cyst, but it can be affected by high protein content, altering the signal. Both CCT and CMR are essential when evaluating complicated cases such as loculated or hemorrhagic cysts [48].

## 9. Challenges and Future Directions in the Diagnosis of Cardiac Masses

Despite advances in diagnostic technologies, the evaluation of cardiac masses faces several limitations and unresolved controversies. Their heterogeneity poses a significant challenge, as distinguishing between benign and malignant tumors, thrombi, and other lesions often requires invasive biopsy procedures that are not always safe, feasible, or justified without a clear prognostic impact. The different imaging techniques, while invaluable, have inherent limitations, including variable sensitivity and specificity, overlapping findings among different lesions, contraindications, and high costs. The lack of universally accepted diagnostic protocols exacerbates inconsistencies in selecting appropriate imaging modalities, resulting in variability in clinical practice and, in some cases, redundant or unnecessary testing. Additionally, the unequal availability of advanced imaging technologies globally highlights disparities in care and raises concerns about the feasibility of implementing standardized diagnostic algorithms worldwide.

These challenges call for a multifaceted approach involving research, collaboration, and innovation [49]. Multicenter prospective studies and international registries are critical for building a robust evidence base, while standardized diagnostic algorithms incorporating cost-effective imaging techniques and biomarkers can improve consistency and accuracy in clinical practice. Collaborative efforts among international multidisciplinary teams of cardiologists, oncologists, radiologists, and surgeons should aim at the development and validation of comprehensive guidelines. Furthermore, integrating artificial intelligence and machine learning tools into diagnostic and prognostic frameworks could significantly enhance precision and decision-making. Ultimately, these coordinated efforts should aim to develop non-invasive diagnostic tools and establish equitable systems to ensure worldwide access to advanced diagnostic resources.

## 10. Conclusions

The important and essential role of different noninvasive imaging modalities is described for the diagnosis of cardiac masses. Imaging protocols are tailored to the clinical problem and patient characteristics, but technology is rapidly evolving. We emphasize the multimodality approach with a combined evaluation of all imaging methods and multidisciplinary teamwork. Clinicians aim to customize the most appropriate imaging technique to the patient’s needs.

## Figures and Tables

**Table 1:** Overview of the different imaging techniques.

	Primary Application	Advantages	Limitations
TTE/TEE	TTE is first line test in all scenarios and may be sufficient in the presence of typical pseudomasses (e.g., crista terminalis) in asymptomatic patients. TEE often required for optimal study of valvular lesions.	Widely available, safe, inexpensive. Allows evaluation of the hemodynamic effects of the mass in terms of systolic and diastolic function, valvular competency. May be implemented with RT3DE or contrast echocardiography in specific settings.	Limited acoustic window in some patients, wider inter-observer variability.
CMR	Non-invasive gold standard for tissue characterization.	Provides detailed information on mass composition, dimensions, vascularization, adjacent structure infiltration, and pre-surgical planning.	Limited availability, long acquisition times, claustrophobia, hemodynamic instability, presence of non-compatible devices. Less accurate than echocardiography for masses <7 mm.
CCT	Complementary or alternative to CMR when the latter is unavailable or contraindicated.	Broad availability, fast acquisition times. Optimal for the study of calcifications and pre-surgical planning.	Ionizing radiations, possible allergic reactions or kidney injury from contrast medium. Less temporal resolution than CMR. Less accurate than echocardiography for masses <4 mm.
18F-FDG-PET	Metabolic evaluation of the mass. Malignant tumors show high 18F-FDG uptake.	Visualization of extracardiac metastases or of distal embolization in the context of IE.	Requires patient preparation, possible false positives from inflammatory of infectious conditions or surgical materials.

TTE: transthoracic echocardiography; TEE: transesophageal echocardiography; RT3DE: real-time three-dimensional echocardiography; CMR: cardiac magnetic resonance; CCT: cardiac computed tomography; 18F-FDG-PET: 18-fluorodeoxyglucose positron emission tomography.

**Table 2:** Cardiac magnetic resonance features of the main intracardiac masses.

MASS	T1-Weighted	T2-Weighted	FPP	LGE	Notes
Thrombus	Acute: ↔/↑ Chronic: ↓	Acute: ↔/↑ Chronic: ↓	Absent	Absent	Typical TI scout pattern *
Myxoma	↔	↑	Minimal/yes	++ Heterogeneous	
Lipoma	↑	↑	Absent	Absent	India ink artifact; ↓ with fat saturation
Rhabdomyoma	↔	↑	Absent	Minimal	
Fibroma	↔/↓	↓	Absent	+++	No early enhancement
Hemangioma	↔	↑	Yes	Minimal	++ early enhancement
Sarcoma	↔	↑	Yes	++ Heterogeneous	
Lymphoma	↔/↓	↑	Yes	Minimal/absent	
Metastasis	↓	↑	Yes	++ Heterogeneous	Melanoma: ↑ T1

* Typical TI scout pattern of thrombi: hyperintensity/isointensity with short TI and hypointensity with long TI, distinct “edged” appearance with a dark rim and central bright zone at intermediate TI [34]. Legend: ↔ = isointense to normal myocardium; ↑ = hyperintense to normal myocardium; ↓ = hypointense to normal myocardium; ++ = increased uptake; +++ = hyperenhancement. FPP: first-pass perfusion, TI: post-contrast inversion time.

## Abbreviations

PCTs: primary cardiac tumors; TTE: transthoracic echocardiography; TEE: transesophageal echocardiography; RT3DE: real-time three-dimensional echocardiography; CMR: cardiac magnetic resonance; LGE: late gadolinium enhancement; CCT: cardiac computed tomography; 18F-FDG-PET/CT: 18-fluorodeoxyglucose positron emission tomography/computed tomography; SPECT: single photon emission computed tomography; LA: left atrium; LAA: left atrial appendage; RA: right atrium; LV: left ventricle; RV: right ventricle; MI: myocardial infarction; TI: inversion time; IE: infective endocarditis; NBTE: non-bacterial thrombotic endocarditis; PFEs: papillary fibroelastomas; PCLs: primary cardiac lymphomas; CMAC: caseous mitral annular calcification; MAC: mitral annular calcification.
